# The influence of gaze direction on time perception: From the perspective of social perception

**DOI:** 10.3389/fpsyg.2022.967603

**Published:** 2023-01-25

**Authors:** Weicong Ren, Xiujuan Guo, Jinfeng Huang, Qingjun Liu, Zhijie Zhang

**Affiliations:** Department of Psychology, Hebei Normal University, Shijiazhuang, China

**Keywords:** gaze direction, duration perception, same-race, other-race, time overestimation

## Abstract

Gazing is important during communication, which is a type of body language that differs from culture to culture. The present study investigated the influence of direct and averted gaze directions on the perceived duration of gazing among same-race and other-race faces. The gaze direction effect, in which direct-gazing faces were perceived to be longer than averted-gazing faces were, was found in the same-race condition but not in the other-race condition. The results could promote our understanding of the underlying mechanism of the gaze direction effect based on the perception of interpersonal social interaction.

## 1. Introduction

Gazing is a type of body language that is extremely important during communication (Kleinke, 1986). It involves interpersonal visual connection. The direction of the gaze (mutual or averted), a crucial medium through which humans can transmit socially relevant information (Macrae et al., 2002), may influence the perceived duration of face images. Appropriate eye contact levels differ from culture to culture; for example, maintaining eye contact during social interaction is a more important principle for Western Europeans than for East Asians (Argyle et al., 1986). Therefore, the perceived durations of gazes may vary for same-race and cross-race faces with various gaze directions.

Many studies have investigated the effect of gaze direction on time perception. Photographs of faces that exhibited direct or averted gazes while expressing anger or happiness were used to investigate the effects of gaze direction on the perceived duration of the presentation of angry and happy expressions in a bisection task. The results revealed that the presentation durations of faces with straight gazes were perceived to be longer than those of faces with averted gazes in the angry expression condition, but not in the happy expression condition.

However, Jusyte et al. (2015) did not find an effect of gaze direction on temporal judgments. The stimuli were faces showing a direct or averted gaze and exhibiting either an angry or neutral affective state. Unlike the study by the participants in Jusyte et al. (2015) were clinical samples of social anxiety disorder and healthy controls. Participants performed a temporal bisection task before and after the stress provocation phase, which induced state anxiety. Therefore, according to Jusyte et al. (2015), in addition to the participants, emotional factors from stimuli may have an effect on the results.

Found the gaze direction effect (straight gazes were perceived to last longer than averted gazes) in angry face conditions only, while Jusyte et al. (2015) found an overestimation effect for angry versus neutral facial expressions, but no gaze direction effect. These inconclusive results suggest that there may be a complicated interaction between emotion and gaze direction. Thus, the inclusion of emotional factors may confuse the investigation of the gaze direction effect on duration perception. In this study, we used neutral faces with direct-and averted-gaze directions to clarify the effect of gaze direction on duration perception.

Thönes and Hecht (2016) found an overestimation of duration for neutral faces in the direct-gaze condition, but it was statistically insignificant with a digitally morphed head. They also explored this effect with naturalistic photographs of different people; however, the pictures they used were a mixture of neutral and emotional faces. Burra and Kerzel (2021) found that participants underestimated the durations for neutral faces in the direct-gaze condition, which was limited to dynamic stimuli in which the gaze appeared to move toward the participant, and it was absent for static pictures. The above studies indicate the absence of a direct-gaze effect on perceived duration for neutral static face pictures. Notably, in the study of Thönes and Hecht (2016), the stimulus used for the neutral condition was a digitally morphed head, which is not a real face. While the stimuli under direct-gaze condition in Burra and Kerzel (2021) were pictures of faces averaged by 30^°^ with eyes gazing straight toward the observer. It is different from the direct-gaze condition in other research (for example, Jusyte et al., 2015; Thönes and Hecht, 2016). It is necessary to explore the effect of gaze direction on duration perception while keeping the experimental operations as consistent as possible.

The pacemaker-accumulator model is usually used to explain the underlying mechanism of time perception, which proposes an internal clock that comprises a pacemaker emitting pulses and an accumulator collecting these pulses (Treisman, 1963; Meck, 1996). Changes in the pacemaker as well as changes in the accumulation can alter perceived duration, which is related to the accumulated pulses. It has been demonstrated that higher levels of arousal are associated with an increased rate of pulse emission (Droit-Volet and Meck, 2007; Mella et al., 2011; Gil and Droit-Volet, 2012), resulting in overestimation of perceived duration. Alternatively, paying attention to the time interval may cause the accumulator to miss fewer pulses from the pacemaker and produce a longer perceived duration (Block and Zakay, 1996; Zakay and Block, 1996; Brown, 1997).

It has been suggested that a direct gaze induces more arousal than an averted gaze (Ellsworth and Langer, 1976; Helminen et al., 2011). As a result, a direct (mutual) gaze could lead to overestimation of subjective time and prolonged duration judgments. Confirmed this hypothesis; however, Jusyte et al. (2015) did not find such an effect. In fact, both studies mentioned emotional faces, but neither assessed the arousal level of the stimuli. Using neutral static face pictures, Burra and Kerzel (2021) found no significant direct-gaze effect. Similarly, however, they did not evaluate the arousal levels of the stimuli. Thönes and Hecht (2016) examined the arousal level of the stimuli but found no significant differences between arousal ratings for direct-and averted-gaze stimuli. It should be noted that they used digitally morphed heads (Experiments 1 and 2) and naturalistic photographs of human faces, mixing photos of celebrities and colleagues (Experiment 3). Thus, the absence of arousal differences in the direct-and averted-gaze conditions may have resulted from the dominant influence of identity information on the arousal level. In view of the above inconsistency of research methods and results, further studies are needed to clarify whether gaze directions without emotional expressions affect the perceived duration of the face pictures, meanwhile, whether the arousal levels of direct and averted gazes play a role in the perceived duration of the faces stimuli.

Besides arousal level, viewing direct and averted-gazing face pictures may induce different perceptions of social interaction, which may affect viewers’ judgments about the duration of presentation of face pictures. A “social interaction” hypothesis suggests that an accurate and precise representation of appropriate mutual gaze duration is of high social relevance (Harrison et al., 2015). Therefore, temporal perceptions of mutual gaze stimuli are expected to be sensitive to small differences in duration. To further manipulate the perception of social interaction, this study included images of other-race faces. The well-known “other-race” effect depicts the tendency to be more proficient with face recognition within one’s own ethnic group compared with other ethnic groups. Poor recognition of other-race faces usually impacts everyday social interactions (McKone et al., 2021), which may play a unique role in time perception (e.g., Liu et al., 2018; Ren et al., 2021). Thus, for Chinese participants, there are differences in the perceived interpersonal interaction information when viewing pictures of same-race and other-race faces, and this difference will help us understand the internal mechanism by which gaze direction affects time perception.

To sum up, in this study, we exploited pictures of faces that exhibited direct or averted gazes from the same as well as other races to investigate the influence of gaze direction on perceived duration. A two-factor within-subject design was used. The independent factors were image type (same vs. other-race faces) and gaze direction (frontal vs. 45° left-side vs. 45° right-side). We evaluated the arousal, valence, and attractiveness of the face pictures to exclude potential confounding factors and to clarify the mechanism of the influence of gaze direction on time perception.

## 2. Materials and methods

This study was approved by the ethics committee of Hebei Normal University. Prior to the experiment, the experimenter explained the procedure to the participants and obtained written informed consent in accordance with the ethical guidelines of the university and the Declaration of Helsinki.

### 2.1. Participants

G*Power 3.1.9.2 software (University of Kiel, Kiel, Germany) was used to calculate the minimum sample size. This indicated that a minimum sample size of 24 was required to obtain 90% power to detect a medium effect of Cohen’s *f* = 0.25. A total of 30 Chinese volunteers participated in the experiment (eight males, M = 24.25 years, SD = 2.19). All participants had normal or corrected-to-normal vision and were naïve to the aim of the experiment. The participants were paid after completing the experiment.

### 2.2. Stimuli

**Figure 1:**
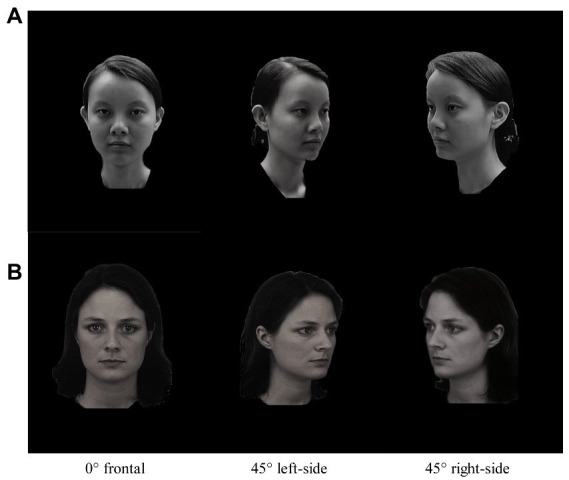
Examples of the stimuli showing three gaze directions. **(A)** Model CF1 from CAS-PEAL (Chinese Academy of Sciences pose, expression, accessory, and lighting; Gao et al., 2008). **(B)** Model FF1 from KDEF (The Karolinska Directed Emotional Faces; Lundqvist et al., 1998).

Chinese and Caucasian face pictures were used as the stimuli. Pictures of Chinese faces were selected from CAS-PEAL (Chinese Academy of Sciences pose, expression, accessory, and lighting; Gao et al., 2008), of which pictures numbered less than 100 and marked with “R1” are allowed to be cited. Caucasian faces were selected from KDEF (The Karolinska Directed Emotional Faces; Lundqvist et al., 1998). Face pictures of eight models were selected from each picture gallery (four males and four females, 20–30 years old), with no obvious distinguishing features, such as glasses, beards, scars, or moles on the face. The face pictures of each model were taken from three different gaze directions: frontal (0°), three-quarters leftward as seen from the participants (+45°), and three-quarters rightward as seen from the participants (−45°). The Chinese faces were the same-race-type stimuli for the participants, and the Caucasian faces were the other-race-type stimuli. A total of 48 pictures of Chinese and Caucasian faces were used. All images were shown on a grayscale with a neutral facial expression. Examples of the stimuli are shown in Figure 1. Adobe Photoshop CS5 software (Adobe systems, San Jose, CA, United States) was used for image processing. The face, hair, and neck of the models in the pictures were retained, and other external features were removed. All images were grayscale, with consistent contrast and brightness. Each image measured 360 × 480 pixels.

Rating task: The attractiveness, valence, and arousal level of the facial stimuli were rated on a nine-point Likert scale. Forty-seven undergraduate students (five males) who would not attend the subsequent experiment participated in the rating task. The participants indicated their affective reactions to the direct-gaze and averted-gaze stimuli, with each face picture presented for 5 s. The presentation order of the stimuli was counterbalanced between participants.

### 2.3. Procedure

E-Prime software 2.0 was used for the programming and presentation of stimuli. Stimuli were presented on a CRT 17″ monitor (1,024 × 768-pixel screen resolution; 85 Hz refresh rate).

The participants completed the experiment alone in a quiet, well-lit room. Before the experiment, the participants were required to sit in front of the computer at a distance of approximately 50 cm from their eyes to the monitor. Participants were then informed of the instructions.

**Figure 2:**
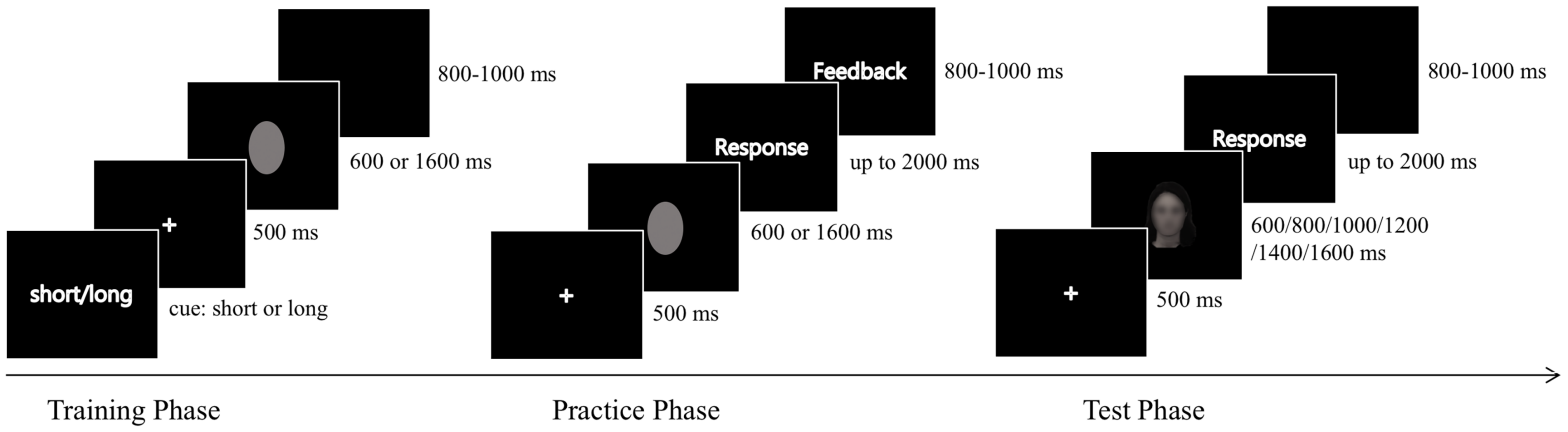
Schematic flow of the experiment. In the training phase, a 500 ms fixation followed a cue indicating the short or long standard duration (presented as a gray ellipse) that would appear subsequently. A blank screen presented at a random time between 800 and 1,000 ms before the next trial. In the practice phase, a gray ellipse presented for either 600 or 1,600 ms appeared after a 500 ms fixation. The participants were required to classify the duration of the ellipse into a short or long standard interval. Thereafter, feedback was presented for 800–1,000 ms. In the test phase, the face pictures replaced the gray ellipse and were presented as probe durations. A blank screen replaced the feedback in this stage.

The time-bisection task consisted of three phases: training, practice, and test (Figure 2). In the training phase, an ellipse was presented with two standard durations (600 or 1,600 ms), with each duration repeated three times. In the practice phase, an ellipse was presented for 600 or 1,600 ms after a 500 ms fixation, which followed by a blank display for participants to judge which standard duration (“short” or “long”) was used for the just-presented ellipse by pressing the “F” or “J” key. The response keys were counterbalanced across the participants. Feedback (“true” or “false”) followed each response. Each participant was given successive blocks of 10 trials, consisting of five short standard duration trials and five long standard duration trials. The trial order was randomized across participants and across blocks. The practice phase was terminated when the observers completed 10 consecutive correct responses. Most participants completed the practice phase after 10–15 practice trails.

In the test phase, each test stimulus was presented with six probe durations (600, 800, 1,000, 1,200, 1,400, and 1,600 ms) after a 500 ms fixation display. Participants were asked to judge whether the probe duration was more similar to the long or short standard duration by pressing the corresponding key used in the training phase. They were required to respond as quickly as possible with a maximum response time of 2 s. Feedback was not provided during this phase. Each face picture was repeated six times in total, and each time, the image was presented with one of the six probe durations. There were four blocks with 12 face pictures in each block, six of which were Chinese faces gazing in different directions. The face pictures within one block were randomized, and the order of the blocks was counterbalanced between the participants. Each participant completed 288 trials. Participants rested every 72 trials, and the entire experiment lasted approximately 30 min.

**Figure 3:**
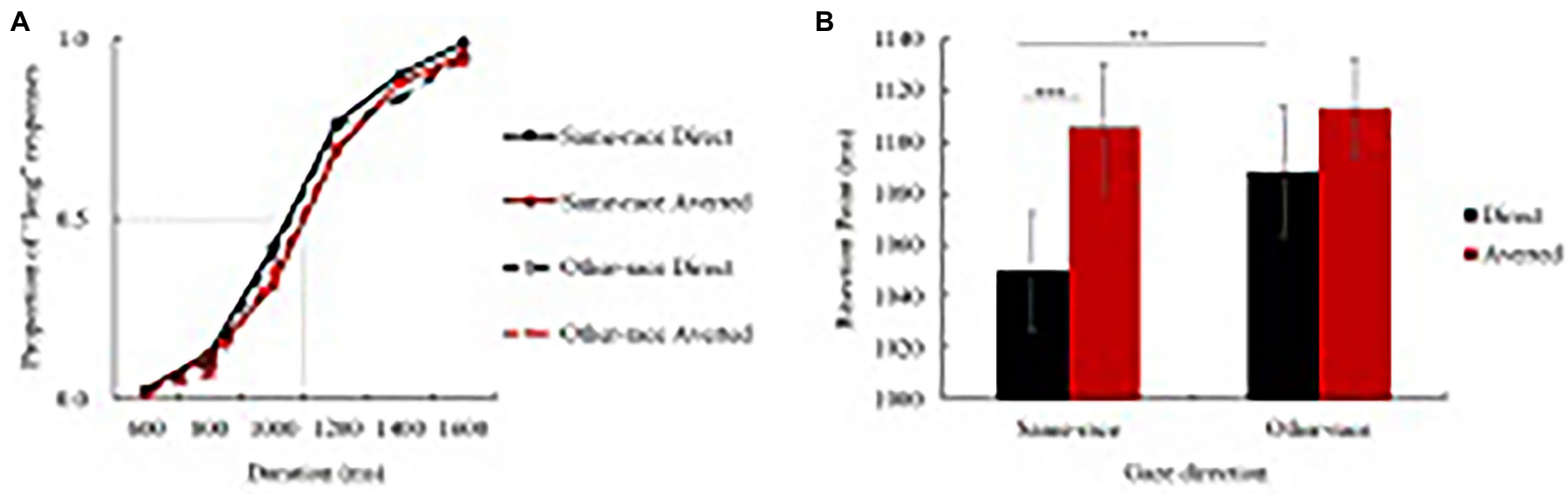
Results of the time-bisection task. **(A)** Psychometric functions fitting the proportion of “long” responses as a function of stimulus duration and gaze direction. The gray dotted line indicates that if the stimuli were perceived as equal to the actual duration, the BP should be 1,100 ms. A left shift of the BP indicates an overestimation of the probe duration, while a right shift demonstrates an underestimation. **(B)** The bisection point (BP) under each type of stimuli condition. **p < 0.01, ***p < 0.001. Error bars represent standard error.

Matlab R2010b was used to calculate the data of the time-bisection task. For each participant, the proportion of “long” responses [p (long)] for each gaze direction and stimulus duration was calculated. The group-averaged psychometric functions are shown in Figure 3A. The bisection point (BP) was then calculated. The BP is the duration at which a participant is equally likely to choose “long” or “short,” or 50% of each. On the cumulative normal distribution curve, the BP is the value of the abscissa corresponding to the point at which the value of the ordinate is 0.5. It reflects accuracy in relation to the veridical middle point, with lateral displacement indicating response bias toward either short or long responses. Specifically, a larger BP indicates an underestimation of the standard time interval, while a smaller BP demonstrates an overestimation of it.

The data of the rating task, as well as the BP were analyzed by means of a repeated-measures analysis of variance (rmANOVA) using JASP 0.16.4.0. The Bayes factors were also calculated, and the model-averaged results (the inclusion Bayes factor, BF_incl_) were reported.

## 3. Results

Table 1 presents the results of the rating tasks. The main effect of image type was significant on arousal level, *F*(1, 46) = 5.029, *p* = 0.03, *η*_p_^2^ = 0.10, with same-race type images having higher arousal levels than other-race type images. The two types of images showed no significant differences in attractiveness, *F*(1, 46) = 0.472, *p* = 0.496, and valence, *F*(1, 46) = 2.186, *p* = 0.146.

**Table 1:** Results of the stimuli assessment task (M ± SD).

Stimuli	Gaze direction	Attractiveness	Valence	Arousal
Same-race face pictures	0° frontal	4.52 ± 1.76	4.94 ± 1.48	4.23 ± 1.66
	45° right-side	4.48 ± 1.85	4.96 ± 1.55	4.13 ± 1.72
	45° left-side	4.52 ± 1.81	4.84 ± 1.47	4.20 ± 1.61
Other-race face pictures	0° frontal	4.47 ± 1.76	4.85 ± 1.54	4.06 ± 1.63
	45° right-side	4.57 ± 1.75	4.85 ± 1.51	4.14 ± 1.59
	45° left-side	4.56 ± 1.72	4.82 ± 1.42	4.06 ± 1.55

The main effect of gaze direction was not significant on attractiveness, *F*(2, 92) = 0.588, *p* = 0.558; valence, *F*(2, 92) = 1.378, *p* = 0.257; or arousal level, *F*(2, 92) = 0.051, *p* = 0.951. The interaction effects of image type and gaze direction were not significant for attractiveness, *F*(2, 92) = 1.676, *p* = 0.193; valence. *F*(2, 92) = 0.363, *p* = 0.697; or arousal level, *F*(2, 92) = 2.225, *p* = 0.114.

Firstly, the BP was analyzed by means of rmANOVA. As shown in Figure 3B, the main effect of stimuli type was not significant, *F*(1, 29) = 3.471, *p* = 0.073, *η*_p_^2^ = 0.107, BF_incl_ = 1.202. The main effect of gaze direction was significant, *F*(2, 58) = 10.746, *p* < 0.001, *η*_p_^2^ = 0.270, BF_incl_ = 123.496. The interaction effect of stimuli type and gaze direction was marginally significant, *F*(2, 58) = 2.861, *p* = 0.065, *η*_p_^2^ = 0.090, BF_incl_ = 2.465. Further analyses showed that there was no significant difference between left and right averted-gaze conditions, so the data were combined and taken as one averted condition, i.e., the other-side gaze condition. For the same-race face pictures, BP in the direct-gaze condition was significantly smaller than that in the other-side gaze direction, *F*(1, 29) = 18.515, *p* < 0.001, *η*_p_^2^ = 0.390. However, for the other-race face pictures, no significant difference on the BP was found between the gaze direction conditions, *F*(1, 29) = 4.087, *p* = 0.053, *η*_p_^2^ = 0.124. For the direct-gaze condition, BP in the same-race condition was significantly smaller than that in the other-race condition, *F*(1, 29) = 8.357, *p* = 0.007, *η*_p_^2^ = 0.224, while for the other-side gaze direction, no significant difference on the BP was found between same-and other-race stimuli, *F*(1, 29) = 0.337, *p* = 0.566.

## 4. Discussion

Eye contact is important during interpersonal interactions and is affected by cultural differences. In this study, we investigated the influence of direct-and averted-gaze directions on the perceived duration of the interaction among same-race and other-race faces. We found that race information affected the duration perception of face images presented in different gaze directions. Specifically, in the direct-gaze condition, the durations of same-race faces were perceived as longer than other-race faces. In addition, within the same-race condition, the direct-gaze faces were perceived to be gazing longer than the averted-gazing faces, and this effect was not found in the other-race condition. The inclusion of other-race face pictures could further advance our understanding of the effect of gaze direction on duration perception from the perspective of social interaction.

The present study used naturalistic human faces with neutral facial expressions to investigate the effect of gaze direction on the perceived duration of gazes using facial pictures. We excluded the potential confusing effect of emotional expressions and found an overestimation of the perceived duration of a direct gaze compared to an averted gaze. Previous studies have used an “arousal” account to explain the result that a direct gaze elongated the perceived duration of presentation of faces compared with an averted gaze. Since the study used pictures of emotional faces, it was concluded that high levels of arousal lead to an overestimation of perceived duration. However, in the present study, we did not find a significant difference in arousal level between the direct-and averted-gaze conditions. Hence, this study did not lend support to the “arousal” account.

The “social interaction” hypothesis suggests that an accurate and precise representation of appropriate mutual gaze duration is of high social relevance because it is necessary and important for recognizing and expressing social behavior (Harrison et al., 2015). Therefore, temporal perceptions of mutual gaze stimuli are expected to be sensitive to small differences in duration. Such a socially cued increase in temporal resolution may accelerate the internal clock. Because more pulses accumulate during a given temporal interval when the inner clock runs faster, the mean estimates should be longer. The “social interaction” hypothesis could explain the overestimation of the durations in the direct-gaze condition in relation to the averted-gaze condition in the same-race face pictures.

The dependent variables (indicators of time perception) should be carefully selected. Jusyte et al. (2015) found no effect of gaze direction, which might be attributed to the indicator used. Jusyte et al. (2015) used the D-prime score to evaluate the time-overestimation effect. First, the individual hit and false alarm rates for the “long” responses in each experimental condition were calculated and subsequently transformed into *z*-scores. The *z*-scores for the neutral facial expressions were then subtracted from the *z*-scores for the angry facial expressions to calculate the D-prime values. Considering that the calculation method is rather complex, the D-prime value may be insensitive to the gaze direction.

The gaze direction effect was found when viewing same-race faces, but it was absent when the stimuli were other-race faces. This suggests that race information mediates the effect of gaze direction on time perception. One of the most prominent features during interactions with members of other ethnic groups is that their faces “all look the same.” Thus, the reduced perceptual sensitivity in other-race condition (Collova et al., 2017), leading to lower perception of social interaction information, may affect the effect of gaze direction. Besides, there may be different “default” processing strategies: configural for same-race faces and featural for other-race faces (Tanaka et al., 2004; Michel et al., 2006a,b, 2007, 2010). Other-race faces may demand more attentional resources for processing than same-race faces because of their low sensitivity, which may interfere with the gaze direction effect. In addition, the participants in the present study had no real interracial interaction experiences. It is possible that viewing pictures of other-race faces does not initiate mental activity related to perceptions of social interaction. Thus, gaze direction had no effect on the duration of presentation of other-race faces.

The influence of race on time perception has been studied with white people as participants (Moskowitz et al., 2015). Contrary to our results, they found an overestimation of the gaze duration of other-race faces. Specifically, it was found that time perception slows when observing the faces of black men. Moskowitz et al. (2015) proposed that heightened arousal causes an overestimation of gaze duration with other-race face pictures. In the present study, participants were Chinese, with Chinese and white faces included as stimuli (same-vs. other-race conditions). The self-rating task demonstrated that the arousal level of same-race face pictures was significantly higher than that of other-race face pictures, which was inconsistent with Moskowitz et al. (2015). Hence, we found that the gaze durations of same-race faces were perceived as longer than that of other-race faces in the direct-gaze condition, in line with the “arousal” account. However, a race effect was not found in the averted-gaze condition, which did not support the “arousal” hypothesis. We suggest that these results could be explained by the “social interaction” hypothesis. In the straight gaze condition, same-race faces may induce an interpersonal interaction mental activity that might be missing or very small in the averted-gaze condition. As a result, only a mutual gaze with same-race faces extended the perceived duration of interactions.

By including other-race face images, we were able to determine that the perceptual sensitivity of the stimuli affects time perception, which can explain the absence of a direct-gaze effect in Burra and Kerzel’s study on static face pictures (Burra and Kerzel, 2021). Notably, with static face pictures, we found a direct-gaze effect in the own-race condition, which is inconsistent with study of Burra and Kerzel (2021). This might be explained by the influence of culture. A direct gaze during social interaction is an important principle for Western Europeans (Argyle et al., 1986), whereas for East Asians, a direct gaze is generally considered impolite. Although we did not find a significant arousal difference between direct and averted-gazing face pictures, it can be speculated that for Chinese people, viewing direct-and averted-gazing faces will produce different mental activities, such as different perceptions of possible interpersonal social interaction. Further studies are required to examine this assumption.

## 5. Conclusion

Gaze direction influences the perceived duration of the face images. This effect was mediated by race: same-race faces, compared to other-race faces, caused overestimation of the duration.

## Data availability statement

The raw data supporting the conclusions of this article will be made available by the authors, without undue reservation.

## Ethics statement

The studies involving human participants were reviewed and approved by The ethics committee of Hebei Normal University. The patients/participants provided their written informed consent to participate in this study.

## Author contributions

ZZ designed and monitored the experiment. WR interpreted the data and drafted the article. XG, JH, and QL collected and analyzed the data. All authors contributed to the article and approved the submitted version.

## Funding

This work was supported by the Social Science Foundation Project of Hebei Province, China (HB21JY024), and the Natural Science Foundation of Hebei Province, China (C2019205282).

## Conflict of interest

The authors declare that the research was conducted in the absence of any commercial or financial relationships that could be construed as potential conflicts of interest.

## Publisher’s note

All claims expressed in this article are solely those of the authors and do not necessarily represent those of their affiliated organizations, or those of the publisher, the editors and the reviewers. Any product that may be evaluated in this article, or claim that may be made by its manufacturer, is not guaranteed or endorsed by the publisher.

## Supplementary material

The Supplementary material for this article can be found online at: https://www.frontiersin.org/articles/10.3389/fpsyg.2022.967603/full#supplementary-material
